# Causes for Withdrawal in an Urban Peritoneal Dialysis Program

**DOI:** 10.1155/2015/652953

**Published:** 2015-04-30

**Authors:** Biruh Workeneh, Danielle Guffey, Charles G. Minard, William E. Mitch

**Affiliations:** ^1^Division of Nephrology, Baylor College of Medicine, Houston, TX 77030, USA; ^2^Dan L. Duncan Institute for Clinical & Translational Research, Baylor College of Medicine, Houston, TX 77030, USA

## Abstract

*Background*. Peritoneal dialysis (PD) is an underutilized dialysis modality in the United States, especially in urban areas with diverse patient populations. Technique retention is a major concern of dialysis providers and might influence their approach to patients ready to begin dialysis therapy.* Methods*. Records from January 2009 to March 2014 were abstracted for demographic information, technique duration, and the reasons for withdrawal.* Results*. The median technique survival of the 128 incident patients during the study window was 781 days (2.1 years). The principle reasons for PD withdrawal were repeated peritonitis (30%); catheter dysfunction (18%); ultrafiltration failure (16%); patient choice or lack of support (16%); or hernia, leak, or other surgical complications (6%); and a total of 6 patients died during this period. Of the patients who did not expire and were not transplanted, most transferred to in-center hemodialysis and 8% transitioned to home-hemodialysis.* Conclusions*. Our findings suggest measures to ensure proper catheter placement and limiting infectious complications should be primary areas of focus in order to promote technique retention. Lastly, more focused education about home-hemodialysis as an option may allow those on PD who are beginning to demonstrate signs of technique failure to stay on home therapy.

## 1. Introduction

Peritoneal dialysis (PD) is an underutilized dialysis modality in the United States, especially in urban centers with diverse patient populations. Overall, excluding patients who may have difficulty performing the procedure (e.g., stroke, poor vision, and previous abdominal surgery), as many as 85% of stage 5 chronic kidney disease (CKD) patients are medically eligible for peritoneal dialysis [1]. However, patients on PD comprise only 10% of the entire end-stage renal disease (ESRD) population and the percentage of African-American and Hispanic ESRD patients treated with peritoneal dialysis has been consistently lower than that of White dialysis patients [2]. Surveys show that only two-thirds of ESRD patients beginning maintenance dialysis are presented with peritoneal dialysis as an option. African-Americans comprise 37% of the ESRD population but comprise only 25% of the patients receiving PD. Hispanic patients comprise 17% of the total ESRD population but only 13% of PD patients [3]. The reasons for this disparity are complex and are the subject of ongoing study.

Several studies have identified the physician's role in dialysis choice; however physician bias is difficult to gauge [4, 5]. One reason that is clear from the data is that if the physician does not believe that patients have sufficient family or social support then they are referred to PD at significantly lower rates [6]. This suggests that the physician's assessment of the probability of technique retention is a major factor in encouraging one modality over another, and presumably this disproportionately affects patients who are resource-poor in urban settings.

When technique failure does occur in PD, it does so at a rate over 40% in the first year [7]. Unfortunately, there have been only a handful of dated reports that have examined this issue in-depth. Previous reports suggest that age, gender, comorbid diabetes, and low socioeconomic factors are independently associated with technique failure [8, 9]. We sought to examine technique survival and the particular reasons for PD withdrawal among incident patients in an urban, racially diverse practice setting near downtown Houston, Texas.

## 2. Methods

Approval from the Human Subjects IRB at Baylor College of Medicine and authorization from the dialysis center (Satellite Health/Wellbound of Houston) were obtained prior to the conduct of this study. Records of all adult patients from January 2009 (when the unit began to recruit patients) to March 2014 were abstracted for demographic information, technique duration, and the reasons for withdrawal. Patients were classified as African-American, Hispanic-Latino, White, Asian, and Native American based on what was indicated on Center for Medicare and Medicaid Services (CMS) reporting forms. ESRD diagnosis was also abstracted from reporting forms. Catheter malfunction, peritonitis, and exit site infection were assessed as well as kidney transplantation. Technique failure was defined as discontinuation of PD for more than 6 weeks, but this did not include patients who were transplanted or recovered renal function.

Patient characteristics are summarized using mean and standard deviation or frequency and percentage. Patients who expired, were transplanted or recovered kidney function were censored in the analysis and all others were considered as treatment failure (events). Kaplan-Meier plots were generated to indicate time to treatment failure, log-rank test was used to compare time to event between groups, and Cox proportional hazards regression was used to evaluate associations between age, race, and gender with risk of treatment failure. Death and transplant were explored as competing risks but did not change the results and were not included in the analysis.

## 3. Results

**Figure 1:**
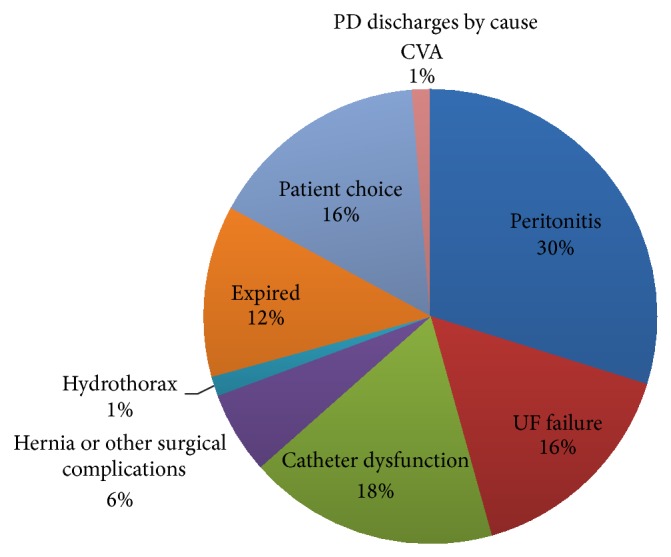


There were 128 incident ESRD patients included in the study and their characteristics are listed in Table 1 and the reasons for PD withdrawal in Figure 1. The principle reasons for PD withdrawal were repeated peritonitis (30%); catheter dysfunction (18%); ultrafiltration failure (16%); patient choice or lack of support (16%); or hernia, leak, or other surgical complications (6%); and a total of 6 patients died during this period. Of the patients who did not expire and were not transplanted, 51% transferred to in-center hemodialysis and 8% to home-hemodialysis at the same center. Not included in the calculation were 12 patients (17% of discharges) who were transplanted and 8 patients (12% of discharges) who changed PD units.

**Figure 2:**
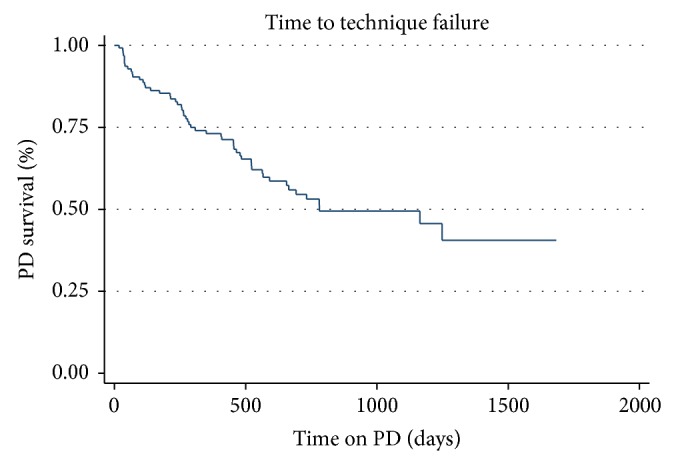


The median technique survival was 781 days (2.1 years) during the study window.  Figure 2 shows the aggregate Kaplan-Meier time to technique failure among all the patients analyzed. Of the 128 patients, a total of 54 patients were classified as technique failure (excluding transplant, renal recovery, and death) during the study window and the mean length of technique survival in this group who withdrew from PD was 344 days.

**Figure 3:**
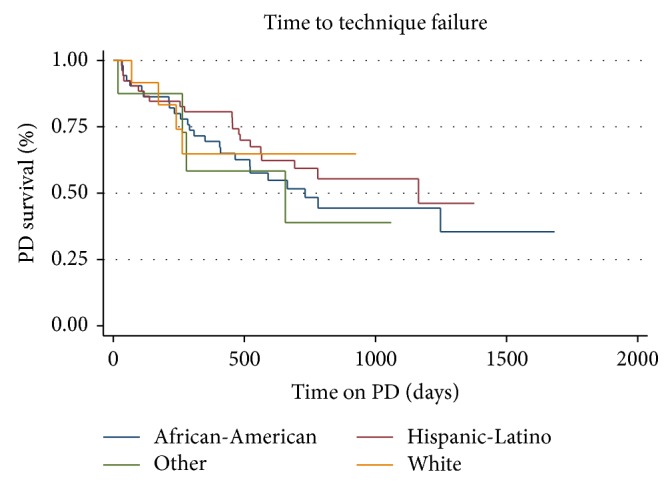


**Figure 4:**
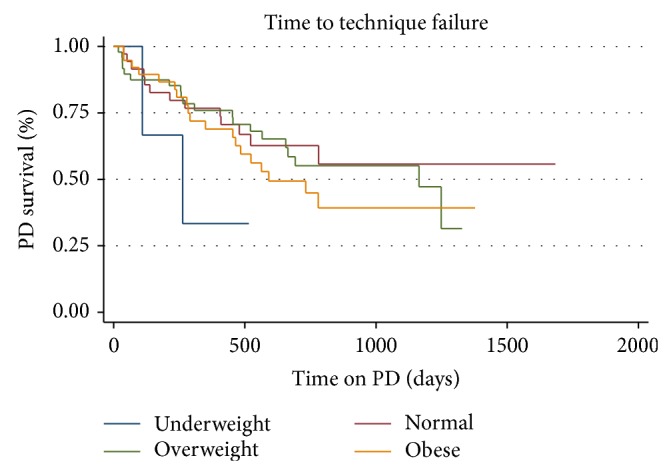


**Figure 5:**
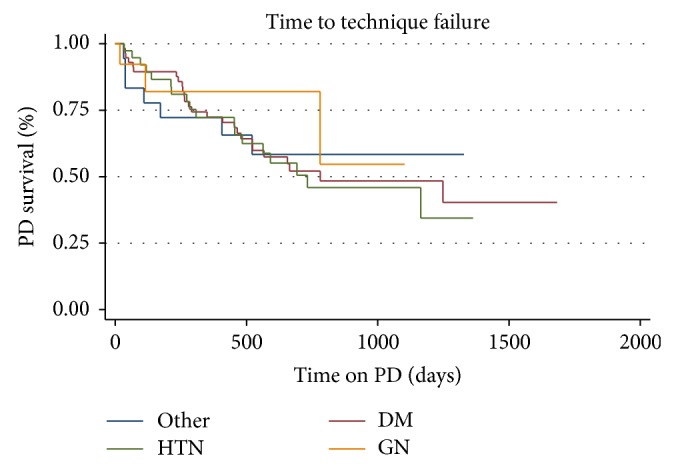


Several factors that may have influenced technique survival were examined. Gender was not associated with time to technique failure (*p* = 0.951). Race is not associated with time to technique failure (*p* = 0.548) when race has 5 categories (African-American, Asian, Hispanic, Native American, and White). When grouping Asian and Native American together race still was not associated with time to technique failure (*p* = 0.7233) (Figure 3). Categories of age by quartiles were not associated with technique failure (*p* = 0.904). BMI by quartiles was also not associated with time to technique failure (*p* = 0.974). When BMI was defined by weight categories (underweight: BMI < 18.5; normal: 18.5–25; overweight: 25–30; obese: >30), it was also not associated with time to technique failure (*p* = 0.3794) (Figure 4). When limited to 3 major categories and “other,” there was no significant difference in survival by cause of ESRD (*p* = 0.885) (Figure 5). From the Cox proportional hazards model individual or combined, none of the variables examined were significantly associated with hazard of technique failure.

## 4. Discussion

In our analysis, peritonitis is the primary cause of transfer from PD, and we acknowledge that although peritonitis is a leading precipitating event for transfer, we speculate that the genuine reason may be patient burn-out, noncompliance, inadequate dialysis, a request based on lifestyle, or a persistent exit site infection. Although our sample size may be too small to make definitive conclusions, factors such as gender, BMI, and ESRD diagnosis did not make a difference in technique survival and African-American and Hispanic patients compare favorably to other groups.

The merits of peritoneal dialysis are apparent, including safety, cost, and quality of life. There are no differences in peritoneal transport characteristics and likelihood of achieving adequacy among varying racial groups [10]. Furthermore, technique survival and the reasons for technique failure are not appreciably different in this population comprised of mostly African-American and Hispanic patients in prior surveys that have been performed [11]. A report by Korbet et al. and Tanna et al. found that PD may be associated with better long-term patient survival in African-Americans [12, 13]. Still other studies found that sociodemographic factors, such as fewer years of education, employment, and US minority status, are associated with lower technique survival [14]. More updated studies with more rigorous methodologies outcomes are needed.

An analysis by DePasquale et al. suggests that there may be differences in the way patients and families of different backgrounds make informed treatment choices, and a better understanding of these differences can help dialysis providers more effectively facilitate decisions related to dialysis choice [15]. In light of the present study and the ones that have been reported previously PD as technique should be supported in all eligible populations.

This study does have its limitations including a relatively small sample size and lack of “exit” surveys in the methodology, which may have enhanced understanding of the results. A regional analysis of incident and prevalent patients in urban PD programs that have a critical mass of patients and that collected center specific data (e.g., physician, nursing experience, etc.) would be an ideal design.

## 5. Conclusions

Our findings suggest that there are a number of causes of PD failure and measures to limit peritonitis should be a primary focus in order to promote technique retention. Other measures such as ensuring patient support to avoid burn-out and coordinating with surgeons to ensure proper catheter function and limiting surgical complications may also contribute to technique retention. More focused education about home-hemodialysis as an option, 8% in our analysis, may present an opportunity for those on PD who are beginning to demonstrate signs of technique failure to stay on home therapy. Lastly, concentrated efforts to educate and offer peritoneal dialysis to Hispanic and African-American could result in a higher proportion choosing PD as a treatment option.

## Figures and Tables

**Table 1:** Description of subjects.

All patients	*N* = 128
Age, mean (SD)	52.0 (13.4)
BMI, mean (SD)	28.0 (5.9)
Race, *N* (%)	
African-American	54 (42%)
Asian	7 (5%)
Hispanic-Latino	53 (41%)
Native American	1 (1%)
White	13 (10%)
Male, *N* (%)	81 (63%)
Diagnosis, *N* (%)	
Failed transplant	2 (2%)
GN	13 (10%)
HIVAN	3 (2%)
HTN	39 (30%)
Nephrotoxicity	1 (1%)
Obstructive nephropathy	2 (2%)
PCKD	2 (2%)
RCC nephrectomy	1 (1%)
Type 1 DM	4 (3%)
Type 2 DM	54 (42%)
Unknown	7 (5%)

## References

[1] Rivara M. B., Mehrotra R. (2014). The changing landscape of home dialysis in the United States. *Current Opinion in Nephrology and Hypertension*.

[2] Martino F., Adibelli Z., Mason G. (2014). Home visit program improves technique survival in peritoneal dialysis. *Blood Purification*.

[3] U.S. Renal Data System (2013). *USRDS 2013 Annual Data Report: Atlas of Chronic Kidney Disease and End Stage Renal Disease in the United States*.

[4] Little J., Irwin A., Marshall T., Rayner H., Smith S. (2001). Predicting a patient's choice of dialysis modality: experience in a United Kingdom renal department. *American Journal of Kidney Diseases*.

[5] Mendelssohn D. C., Mullaney S. R., Jung B., Blake P. G., Mehta R. L. (2001). What do American nephrologists think about dialysis modality selection?. *American Journal of Kidney Diseases*.

[6] Jager K. J., Korevaar J. C., Dekker F. W., Krediet R. T., Boeschoten E. W., NECOSAD Study Group (2004). The effect of contraindications and patient preference on dialysis modality selection in ESRD patients in the Netherlands. *American Journal of Kidney Diseases*.

[7] Chidambaram M., Bargman J. M., Quinn R. R., Austin P. C., Hux J. E., Laupacis A. (2011). Patient and physician predictors of peritoneal dialysis technique failure: a population based, retrospective cohort study. *Peritoneal Dialysis International*.

[8] Pulliam J., Li N.-C., Maddux F., Hakim R., Finkelstein F. O., Lacson E. (2014). First-year outcomes of incident peritoneal dialysis patients in the United States. *American Journal of Kidney Diseases*.

[9] Joshi U., Guo Q., Yi C. (2014). Clinical outcomes in elderly patients on chronicperitoneal dialysis: a retrospective study from a single center in China. *Peritoneal Dialysis International*.

[10] Juergensen P. H., Gorban-Brennan N., Troidle L., Finkelstein F. O. (2002). Racial differences and peritonitis in an urban peritoneal dialysis center. *Advances in Peritoneal Dialysis*.

[11] Raj D. S. C., Roscoe J., Manuel A., Abreo K., Dominic S. S., Work J. (1999). Is peritoneal dialysis a good option for black patients?. *American Journal of Kidney Diseases*.

[12] Korbet S. M., Shih D., Cline K. N., Vonesh E. F. (1999). Racial differences in survival in an urban peritoneal dialysis program. *American Journal of Kidney Diseases*.

[13] Tanna M. M., Vonesh E. F., Korbet S. M. (2000). Patient survival among incident peritoneal dialysis and hemodialysis patients in an urban setting. *The American Journal of Kidney Diseases*.

[14] Shen J. I., Mitani A. A., Saxena A. B., Goldstein B. A., Winkelmayer W. C. (2013). Determinants of peritoneal dialysis technique failure in incident us patients. *Peritoneal Dialysis International*.

[15] DePasquale N., Ephraim P. L., Ameling J. (2013). Selecting renal replacement therapies: what do African American and non-African American patients and their families think others should know? A mixed methods study. *BMC Nephrology*.

